# Supplementary material for: Are carbon emissions trading and green financial instruments synergistic? -Comprehensive quantitative research based on content analysis
Source: PLoS One. 2024 Mar 7;19(3):e0298601. doi: 10.1371/journal.pone.0298601 (PMC10919601; doi:10.1371/journal.pone.0298601)
Supplement: S1 Data — (ZIP) [file pone.0298601.s001.zip › Data/地级市Z_F工作报告.zip!/░▓╗╒/░▓╟∞╩╨/└┤╫╘╣1⁄2╓┌║┼í╛┬φ┐╦╩2╛▌═°í┐_2017.docx]

政府工作报告

—2017年1月9日在安庆市第十六届人民代表大会第七次会议上

市长  陈冰冰

各位代表：

现在，我代表市人民政府，向大会报告政府工作，请予审议，并请市政协委员和其他列席人员提出意见。

一、2016年工作回顾

刚刚过去的2016年，全市上下在中共安庆市委的坚强领导下，全面贯彻党的十八大和十八届三中、四中、五中、六中全会精神，深入贯彻习近平总书记系列重要讲话特别是视察安徽重要讲话精神，认真落实省委、省政府决策部署，全面打响产业转型升级、棚户区改造、精准脱贫、重大基础设施建设、增强县域经济实力“五大攻坚战”，着力稳增长、促改革、调结构、惠民生、防风险，较好完成了全年目标任务，实现了“十三五”良好开局。

**——经济保持平稳较快发展。**预计全市生产总值超过1500亿元，增长8%以上。全社会固定资产投资、规模以上工业增加值、社会消费品零售总额分别增长10%、8.5%、11.5%。财政收入267.6亿元，增长8.2%。节能减排任务全面完成。

**——产业升级迈出新步伐。**战略性新兴产业产值增长12%，高于工业增幅3个百分点以上。高新技术产业增加值增长24%，新认定国家级高新技术企业60家。第三产业占比提高1.6个百分点。

**——重大基础设施建设取得重要进展。**安庆成为全国“八纵八横”高铁网络节点城市，合安高铁全线建设，望东长江大桥建成通车，区域性交通枢纽地位进一步凸显。下浒山水库全面开工。

**——人民生活进一步改善。**城镇常住居民人均可支配收入26429元、增长8%，农村常住居民人均可支配收入10834元、增长8.5%。新增城镇就业6.1万人，城镇登记失业率3.4%。127个贫困村出列，13.3万贫困人口脱贫。改造棚户区243万平方米，1.7万户居民搬入新居。

一年来，我们主要做了以下工作：

（一）加快推进调结构转方式促升级。完善产业支持政策，大力发展战略性新兴产业，改造提升传统产业，推动存量向产业中高端、增量向中高端产业迈进。**促进工业经济稳定增长，**新增规模以上工业企业166家，完成投资超过800亿元，江淮、安达尔新能源汽车整车下线，晨兴智能机器人、曙光丁辛醇等项目竣工投产，赛富环新电池包等重大项目开工建设。**推动科技创新能力升级，**新增院士工作站1家、博士后工作站2家、省级以上技能大师工作室3家、科技企业孵化机构5家、工业设计中心6个、企业技术中心11个；引进高层次科技人才创新创业团队10个，科技成果转化105项；发明专利申请量1万件、授权量3005件；新增国家地理标志保护产品1个、中国驰名商标3件、国家地理标志证明商标6件，华茂集团荣获省政府质量奖。**支持服务业转型发展，**八佰伴、新城吾悦等城市综合体投入运营；阿里巴巴跨境电商综合服务中心入驻安庆，电商园区基本实现县域全覆盖，快件发送量3100万件，增长95%；潜山、岳西、太湖、宜秀获批创建国家全域旅游示范区。**加快农业产业化步伐，**基本建成“五个百万”基地，新增省级以上农业产业化龙头企业12家、示范农民专业合作社20家、家庭农场24家；耕地流转率49.6%，居全省前列；太湖成为全国农产品质量安全示范县。

    （二）全面加强城乡规划建设管理。坚持以人为本，突出规划先行，加快新型城镇化建设。“多规合一”列入省级试点，城市空间发展规划编制完成。**加强中心城市建设管理，**新建和改造湖心中路、集贤南路等24条道路，外环北路提前8个月建成通车，完成合安高速安庆出口北移迁建工程；康熙河环境综合整治景观工程完工，北部新城污水处理厂完成主体工程；建设新能源汽车充电站67个、充电桩1500根，新增公交线路3条、添置新能源公交车86辆；成功创建全国国土资源节约集约模范市、全国绿化模范城市；大力创建全国文明城市，改造老旧小区25个，完成“五化”建设项目57个，城市综合治理联勤执法全面实施。**加快县城和中心镇建设，**明确县城定位，完善功能配套，扎实推进“三治三增三提升”，人居环境进一步改善，潜山县源潭镇等14个镇成为全国重点镇，岳西县温泉镇成为首批中国特色小镇。**深入推进美丽乡村建设，**开展64个乡镇政府驻地建成区环境整治，建成71个省级中心村，潜山、宜秀整县推进工程深入实施，怀宁县三铺村等3个村入选全国绿色村庄。**改善农村生产生活条件，**改造农村危房1.4万户，解决13万农村人口饮水安全问题，除险加固43座小型病险水库，完成农村道路畅通工程4375公里，建成高标准基本农田28.8万亩。

（三）持续深化改革扩大开放。**加强重点领域改革，**加快推进“去降补”，减免各项税费28.5亿元，城区商品房去化周期由16个月降至4.3个月；加快推进“放管服”，“五证合一、一照一码”商事制度改革全面实施，深入推进大众创业、万众创新，新登记企业1.2万户；加快推进“全创改”，实施市属国资国企改革，健全“1+4”监管机制；创新投融资体制机制，组建天使、股权、产业招商等7支基金，争取国家专项建设基金项目209个、128.3亿元，集友新材料股份有限公司IPO过会待发，新增“新三板”挂牌企业8家；加快推进农村综合改革，完成农村土地确权登记颁证，土地承包经营权抵押贷款、集体资产股份制改革试点稳步推进。**不断扩大对外开放，**B型保税物流中心主体工程竣工；中远海运集团入驻安庆；安庆港货物吞吐量2200万吨、增长16%，集装箱吞吐量5.8万标箱、增长13%，天柱山机场旅客吞吐量突破30万人次、增长39%，铁路发送旅客量超过300万人次、增长300%；成功举办闽浙赣皖福州经济协作区市长联席会议、亚布力青年论坛年会；与加拿大汉密尔顿、俄罗斯切博克萨雷、英国伍尔弗汉普顿结成国际友好城市；环新集团成功并购德国一家行业领军上市公司，山城集团等13家企业“走出去”积极开展国际合作。

（四）扎实增进人民群众获得感。全面完成33项民生工程。精准脱贫攻坚战在明确“四大路径” 、制定“五大计划” 、推进“七个全覆盖”的基础上，圆满完成了年度目标任务。全力推进棚户区改造，全面推行“房票制”，在改善群众居住条件和城市面貌上迈出了新步伐。成功创建国家公共文化服务体系示范区，“黄金周·黄梅戏”品牌影响力扩大，《大清名相》全国巡演。扎实推进国家中小学教育质量综合评价改革实验区建设，实施城区教育管理体制改革，推进名校集团化办学，全面推行校长教师交流制度，怀宁、潜山、望江有望通过国家义务教育发展基本均衡县验收；安庆师范学院更名为安庆师范大学，安庆职业技术学院获准建设省级地方技能型高水平大学，安庆医专与安徽医科大学开展合作办学。加快医药卫生体制改革，推进分级诊疗，开展省级县域医共体建设试点；稳妥实施全面两孩政策。成功举办市第十三届运动会、第二届市民运动会，成功承办全国古典式摔跤比赛，市体育场馆实现市场化运营。加快推进养老服务市场化，实施“小而美”等社区养老模式，城区居家养老服务设施全覆盖。桐城、宿松、迎江成功创建全国科普示范县。深化“平安安庆”建设，加强社会治安综合治理，建成天网工程二期，刑事案件、治安案件立案数分别下降18%、33%，人民群众安全感、满意度分别提高0.6和3.5个百分点；实施阳光信访、责任信访、法治信访，信访形势稳定好转；加强法律援助、人民调解、社区矫正工作；开展安全生产执法能力建设年活动，安全生产事故起数、死亡人数分别下降18.7%、20.5%；深入创建食品药品安全城市；大气、水污染防治任务全面完成；开工建设安庆石化危化品码头及油气输送管线迁建工程，完成铁路道口平改立51处。建成开放市烈士陵园。切实保障妇女、儿童、老年人、残疾人权益，不断加强民族宗教、外事侨务工作，国防动员、人民防空、国家安全、地震、气象、涉台、保密、档案、地方志、红十字、关心下一代等工作取得新进展。

（五）奋力夺取防汛抗洪和灾后重建的全面胜利。2016年汛期，我市遭遇历史罕见的特大洪涝灾害，在省委、省政府的坚强领导下，我们坚持人民生命至上，快速应对、全面动员，果断决策、靠前指挥，人民解放军、武警官兵和民兵预备役人员不畏艰险、敢打硬仗，广大党员干部冲锋在前、勇挑重担，人民群众和社会各界齐心协力、同舟共济，防汛抗洪抢险救灾取得重大胜利。全市共投入防汛抢险资金8.5亿元，紧急转移安置群众27.4万人，解救洪水围困群众7.3万人，修复水毁水利工程2.1万处、道路820公里，改补种农作物130万亩，5900余户、1.6万余间水毁房屋完成重建。防汛抗灾和灾后重建的全面胜利，进一步升华了不畏艰难、顽强拼搏、众志成城、无私奉献的抗洪精神，为全面推进各项事业发展提供了强大精神动力。

（六）切实加强政府自身建设。深入开展“两学一做”学习教育和“讲看齐、见行动”学习讨论，严守政治纪律和政治规矩，增强政治意识、大局意识、核心意识、看齐意识特别是核心意识、看齐意识，坚决维护党中央权威、坚决维护习近平总书记党的领导核心地位。扎实推进法治政府建设，实现重大事项合法性审查和社会稳定风险评估全覆盖，组织群众公议行政处罚案件409件。自觉接受人大法律监督和政协民主监督，市人大代表建议、市政协委员提案办复率均为100%。建立统一的市县公共资源交易平台，招投标和公共资源交易机制进一步完善。加大行政监察和审计监督力度，加强机关效能建设，干部作风进一步好转，政务服务环境进一步改善。认真落实巡视整改意见，建立健全了一批规章制度和长效机制。

各位代表！过去一年的成绩来之不易。这是省委、省政府和市委坚强领导的结果，是市人大、市政协和社会各界关心支持的结果，是全市人民共同奋斗的结果。在此，我代表市人民政府，向全市广大工人、农民、知识分子、干部、驻宜解放军指战员、武警消防官兵和政法干警，向各民主党派、各人民团体和社会各界人士，向关心支持安庆建设发展的中央和省驻宜单位、海内外友好人士，向在我市创业的投资者、建设者，表示崇高的敬意和衷心的感谢！

在肯定成绩的同时，我们清醒地认识到：我市工业化水平还不够高，战略性新兴产业比重不大，在新的龙头性重大项目上尚未突破；城镇化步伐还不够快，基础设施建设仍有不少欠账，基本公共服务尚未实现优质化均等化，县域经济整体实力不强，脱贫任务亟待全面攻坚；思想解放、对外开放程度还不够到位，以人才为核心要素的创新资源不足，一些重点领域和关键环节的改革仍需深化，体制机制活力需要进一步释放；少数干部的担当精神、执行能力、工作作风等还不能适应新形势新任务的要求，严厉严肃问责也不够。我们将采取有效措施，认真加以解决。

二、2017年重点工作

今年是实施“十三五”规划的重要一年，是贯彻落实省市党代会精神、实施五大发展行动计划的开局之年，做好今年工作意义重大。总体要求是：全面贯彻党的十八大和十八届三中、四中、五中、六中全会精神，深入贯彻习近平总书记系列重要讲话特别是视察安徽重要讲话精神，坚持稳中求进工作总基调，深入推进供给侧结构性改革，围绕省委、省政府关于安庆建设现代化区域性中心城市的战略决策，按照市委“四个强市”“五大攻坚战”“六大工程”的总体部署，大力实施创新、协调、绿色、开放、共享五大发展行动计划，探索后发地区创新发展新模式，形成城乡一体化发展新格局，实现经济生态化和生态经济化新态势，构筑内陆开放新高地，建设人民幸福社会和谐新家园。

经济社会发展的主要预期目标是：全市生产总值增长8.5%，财政收入增长8%，社会消费品零售总额增长12%，全社会固定资产投资增长11.5%，外贸进出口增幅达到全省平均水平，城镇、农村常住居民人均可支配收入分别增长8.5%和9%，居民消费价格涨幅控制在3%左右，城镇登记失业率控制在4.2%以内，脱贫攻坚、节能减排、安全生产完成年度目标任务，社会大局保持和谐稳定。

围绕实施五大发展行动计划，重点做好以下工作：

（一）坚持创新发展，构建产业新体系

打好产业转型升级攻坚战，推进理念创新、制度创新、系统创新，科学定位园区主导产业，做好顶层发展规划，推进园区管理、行政审批、人事薪酬以及包括容错机制在内的考核制度等改革，把科研院所、龙头企业、土地供给、资本支持、人才团队、公共产品、公共服务设施等优势优质资源集成到专业化的园区上，做足主导产业壮链、补链、延链文章，实现全产业链发展，争做全省“四新”产业发展示范市和传统产业转型升级示范市。

**形成市经开区主导产业核心竞争力**。立足于打造国内领先的新能源汽车和重要的智能装备制造产业基地，编制两大主导产业顶层规划。至少引进2家国家级科研院所建立产学研合作平台、成立1支基金、推进1家公司上市、引进3个行业领军人才团队，直接融资20亿元以上。启动新能源汽车全产业链发展，安庆江淮新能源汽车单班达产，赛富新能源电池投产达产，开工建设跃迪新能源汽车项目，汽车零部件产业园初具规模。壮大智能装备制造产业，以建设轨道交通生产基地为主攻方向，争取国家批准建设跨座式单轨轨道交通，加快引进全国知名轨道交通装备制造企业，谋划发展轨道交通装备制造产业；富士康全电注塑机、五轴联动加工设备实现系列化生产，建成中船海工装备等项目。

**积极争创国家级高新区**。市高新区要以争创工作为引领，抓住建设省级知识产权示范园区的机遇，至少新增1家国家级知识产权优势企业、1家企业通过知识产权管理规范认证、10家企业拥有有效发明专利；至少引进1家国家级科研院所建立产学研合作平台、成立1支基金、推进1家公司上市、引进2个行业领军人才团队，直接融资10亿元以上。聚焦医工医药编制产业规划，开工建设恩瑞特、奇创、诺詹药业等项目，建成千辉原料药等项目，启动山口片区建设，致力打造全省领先的医工医药产业发展基地。实施化工产业中高端发展，开工建设蓝蓝树脂涂料、新华胺基甲酸酯等项目，建成投产中玺超高分子量聚乙烯、虹泰聚酰胺树脂等项目，加快推进化工新材料产业集聚发展基地建设。

**着力打造“筑梦”新区**。在现有智慧产业园的基础上，围绕创新发展编制功能规划，以新产业、新技术、新模式、新业态为主攻方向，以市场化运作的创意交流、技术合作、资本对接、成果转化、企业孵化为典型特征，以总部经济集聚为重要抓手，打造创新创业新平台、产业引导新基地、经济发展新引擎，建设中西部地区创新发展的示范区。

**建设各具特色的县域专业化园区**。围绕首位产业编制发展规划，打造一批战略性新兴产业和高新技术企业集聚发展基地。桐城市重点发展智能制造和节能环保产业，怀宁县重点发展新材料和高端装备制造产业，潜山县重点发展医药健康和文化旅游产业，岳西县重点发展大健康和大数据产业，太湖县重点发展功能膜新材料和文化旅游产业，望江县重点发展新能源汽车和纺织服装产业，宿松县重点发展电子信息和纺织服装产业，迎江区重点发展现代服务业，大观区重点发展精细化工产业，宜秀区重点发展食品加工产业。每个县（市）区至少成立1支基金、推进1家公司上市、引进1个行业领军人才团队。

（二）坚持协调发展，推进城乡一体化

推行“多规合一”，坚持“五个统筹”，建设宜业、宜居、宜游、宜购、宜养的“五宜”城市。

**精心打造美好大宜城**。着力完善立体交通体系：推进安庆西高铁枢纽站规划建设，改造升级天柱山机场航站楼、停机坪，做好新机场建设前期工作；谋划长风港二期工程建设，完成五里庙码头升级改造；加快外环西路建设，实现市区主干道与外环路顺畅对接，改造提升德宽路、龙山路、菱湖南路等15条老城区主次干道；做好高铁、港口物流园的规划。着力更新城市人居与人文环境：全面实施绿化亮化提升工程，改善城市形象；推进人民路以南历史文化街区连片保护性开发，呈现城市客厅和城市阳台，促进文商旅融合发展；实施江河湖水系贯通工程，开展“一纵两横”水系治理；推进石塘湖备用水源地及东大湖、西小湖截污工程建设；建成城西污水处理厂二期主体工程。着力推进公共服务优质化均等化：推进安庆医专与安徽医科大学、黄梅戏艺术学院和桐城师专与安庆师范大学合作办学；以安庆职业技术学院为龙头，整合职业教育资源，推动集团化发展，加快建设技工大市；全面启动安庆一中龙山校区等11所新城区学校建设；市立医院东院区、中医院北院区投入运营，市一院龙山院区加快建设；谋划推进医养结合中心、健身公园、会展中心、书城综合体等公共服务设施建设，加快市博物馆建设；改建8个菜市场，完善社区便利店、物流配送等便民设施；新开5条公交线路，新增80辆新能源公交车，建设秦潭湖、红水塘等4个公交枢纽站，开通智能交通运营系统，争创省“优先发展公共交通示范城市”。着力加强城市管理：推进城市管理执法体制改革，整治占道经营、露天烧烤与车辆乱停乱行，规范店招店牌，加强广告设置管理，实施综合清扫、垃圾清运、公厕管护、停车及市容管理等环卫一体化项目，积极争创全国文明城市。

**不断壮大县域经济实力**。打好增强县域经济实力攻坚战，实施县县有上市公司工程，利用好国定贫困县“绿色通道”政策，加快推进企业股改，新增5家以上企业报会、10家以上企业在“新三板”挂牌。实施县县与国家级科研院所合作工程，每个县（市）区至少引进1家国家级科研院所建立产学研合作平台。实施龙头企业培育和引进工程，每个县（市）区至少新增2家纳税千万元以上、5家纳税五百万元以上的龙头企业。下大力气振兴实体经济，努力降低各类交易成本，积极引导企业实施技改，注重加强品牌建设，切实增强企业的市场竞争力。扎实推进农业供给侧结构性改革，推动一二三产融合发展，大力发展农产品深加工业，支持海吉星农产品物流园、宜秀食品工业园、中国供销太湖农产品物流园、宿松新发地农产品和岳西大别山茶叶大市场等一批涉农项目。深化农村综合改革，完善农村土地“三权”分置办法，推进农村集体资产股份合作制改革试点，加快农村产权交易市场建设。

**努力提高新型城镇化水平。**加强对各县（市）区城乡建设的指导，推动农村人口向城镇集中。桐城要围绕建设安庆与合肥之间的卫星城市，打造现代化历史文化名城；怀宁要围绕发展交通枢纽型经济，加快与主城区同城化发展；潜山要围绕建设山水田园城市，深度谋划县改市后的功能提升；岳西要围绕生态优先、绿色发展，打造秀美、精致、宜人的特色山城；太湖要围绕建设全域旅游示范区，提高城镇的承载能力；望江要围绕建设滨江襟湖港口城市，加快产城融合发展；宿松要围绕建设生态工贸型城市，打造皖西南重要门户。布局风情小镇，每个县（市）区谋划建设3个以上特色小镇，突出产业、功能、形态建设，突出以产业为主导、以企业为主体，突出创新创业与企业协同，集聚发展要素，拓展经济增长新空间。全面建设美丽乡村，推广潜山整县推进模式，以农村人居环境整治为重点，实施57个美丽乡镇、120个中心村建设，实现乡镇政府驻地建成区环境整治全覆盖。

（三）坚持绿色发展，建设生态文明示范区

采取工程措施，加强污染防治，推动低碳发展，开展生态修复、城市修补，争创国家环保模范城和环境污染第三方治理试点城市。

**抓好环境污染治理工程。**完成水、土、气污染防治年度目标任务。全面实施重点行业企业清洁生产改造，完成水泥、石油化工等行业烟气脱硫脱硝设施建设，推进安庆石化热电超低排放改造，取缔主城区20吨以下工业燃煤锅炉，淘汰黄标车4800辆以上，实现建筑扬尘治理全覆盖。加强水污染防治，基本完成城区黑臭水体治理；实施污水处理提标工程，推进城区污水厂网一体化建设，建成桐城南部新城、太湖城东污水处理厂。规划建设主城区静脉产业园。深入推进农业面源污染治理，积极争取农作物秸秆综合利用国家试点。

**实施功能区建设工程。**探索大别山区水环境生态补偿机制，推动大别山区域工业园区绿色化改造。以“共抓长江大保护”的理念，编制长江岸线保护性开发和利用规划，建立市县乡三级“河长制”。实施退耕还林、长江防护林建设等重点工程，开展增绿增效行动。推动岳西、太湖、潜山、宜秀创建国家级生态文明建设示范县，其他各县（市）区创建省级生态文明建设示范县。

**推进绿色产业发展工程。**编制全域旅游规划，以标准化要求、国际化视野，促进文旅融合发展，逐步实现统一标准、统一策划、统一线路、统一品牌、统一营销，增强安庆旅游的“黏性”；推动天柱山、文博园等龙头企业实施股份制改造，禅源太湖旅游区成功创建5A级景区，加强岳西旅游景区的基础设施建设，优化城区“一日游”精品线路，全面提升“宜游宜秀”建设水平。编制大健康产业规划，打造大健康产业发展基地，支持发展医养结合、健康食品、体育健身、高端医疗等产业，加快推进岳西国际文化养生产业园、太湖安吉特休养中心等建设。编制新能源产业规划，大力推动清洁能源项目建设，加快桐城抽水蓄能电站筹建步伐，积极推进岳西抽水蓄能电站的前期工作，启动建设宜南五县天然气支线管网工程，全市风电装机总规模达到40万千瓦、光伏发电装机容量突破20万千瓦。

（四）坚持开放发展，提高经济外向度

以大通道建设为前提，拉动腹地对外连接，实现与先发地区无缝对接；以大平台为载体，创新合作模式，建设一批“园中园”；以大通关为保障，优化服务环境，提高便利化水平；以“引进来走出去”为媒介，推动海外并购和产能输出，加强国际经济合作。

**建设大通道。**打好重大基础设施建设攻坚战。加快合安九高铁建设，推进武安杭、宿望宣、六安景、北沿江铁路等项目前期工作。开工建设德州至上饶高速桐城段、岳武高速东延线及安庆联络线；加快安庆至桐城、安庆至望江、潜山至太湖、桐城至小关、高河至黄墩一级公路建设，开工建设安庆至枞阳、太湖至望江、望江至宿松一级公路，实施农村道路畅通工程2200公里。启动宿松王家洲公用码头建设，推进岳西通用机场建设。

**构建大平台。**积极争创综合保税区，建设汽车整车进口等口岸，争取国家将长风港作业区、皖河农场作业区纳入对外开放范围，安庆港综合吞吐能力提高30%以上。积极推进与苏浙沪等先发地区、港澳台、外资企业共建产业园区，每个县（市）区至少建设一个“园中园”。推进电商安庆建设，启动建设区域性快递分拨中心。

**优化大通关。**推进以“负面清单”管理为核心的投资管理体制改革、以贸易便利化为重点的贸易监管制度改革。推动电子口岸平台建设，实行“一站式作业”通关模式，建成B型保税物流中心“一站式”服务大厅。

**加快引进来走出去。**发挥北京、上海、广东、台湾招商平台作用，大力招优引强，力争引进亿元以上项目240个，其中10亿元以上项目20个。鼓励企业开展跨境合作，拓展国际市场，在“一带一路”沿线国家建立基地。支持职业院校与国际实用型大学合作，培养一批具有国际通用职业资格证书的高技能人才。借鉴先发地区做法，筹建外资企业审批、外籍人士生活休闲的综合服务中心。加强国际经济文化交流，新增2个以上国际友好城市。

（五）坚持共享发展，增强群众幸福感

补齐发展短板，提高城乡居民收入水平，健全社会保障制度，完善社会治理机制，推进小康安庆、平安安庆、健康安庆、信用安庆、智慧安庆建设。

**推进小康安庆建设。**按照在全面小康路上决不让一户一人掉队的总要求，打好精准脱贫攻坚战，开展“精准脱贫全面攻坚年”行动，坚持“六看六确保”，完善“能脱贫不返贫”长效机制。推进产业下沉，把经济组织建在村上，因地制宜发展“经济组织+贫困户”模式，稳定困难群众财产性、工资性、经营性收入的来源；切实发挥政策兜底作用，保住基本、兜住底线；选准用好村级带头人，带领群众脱贫致富奔小康；调动各方力量参与扶贫开发，形成大扶贫格局。强化责任落实，严肃追责问责，坚决杜绝“两该两不该”现象。加大财政投入，确保完成民生工程建设任务。完善更加积极的就业政策，实现零就业家庭动态“清零”；推进医保、低保城乡标准合一；加大特困群体和城镇困难家庭帮扶力度，做好“救急难”工作；加强房地产市场调控，促进房地产业健康发展，实现城镇常住人口住房保障应保尽保；打好棚户区改造攻坚战，完成城区棚户区改造300万平方米、老旧小区改造14个；完成农村危房改造1万户，解决8万农村人口饮水安全问题；加快推进潜山500千伏变电站建设，改造升级农村电网。实施第三期学前教育三年行动计划，推进“全面改薄”工程，实现国家义务教育发展基本均衡县全覆盖，完善中小学布局，深化集团化办学，打造全国基础教育名市。加强市县乡村四级公共文化服务设施建设，推进戏曲和高雅艺术进校园，办好黄梅戏展演周，实现文化惠民乐民。加强全民科学素质建设，做好妇女儿童、老年人和关心下一代工作，完成残疾人精准康复任务，加强民族宗教、外事侨务和涉台工作。

**抓好平安安庆建设。**争做全省“一规四补”示范市，推进防洪保安体系建设，开展华阳河流域和菜子湖、武昌湖水系综合治理，建设57座小型水库除险加固工程，开展11条中小河流治理，建成破罡湖闸站，完成下浒山水库大坝建设，加快补齐防汛抗旱短板，变水害为水利。开展安全生产改革发展年活动，建设安全发展示范城市，完成安庆石化危化品码头及油气输送管线迁建工程，推进区域性危化应急救援基地建设。创新社会治理，学习借鉴“枫桥经验”，推广“六尺巷”调解法，加强信访工作，完善矛盾纠纷多元化解机制，加快市县乡村四级综治中心建设，健全立体化、信息化社会治安防控体系，提升突发事件应急处置能力。推动军民深度融合发展，加强国防动员、国防教育、兵役征集、人民防空和民兵预备役工作，大力开展“双拥”共建活动。进一步做好地震、气象、科普、统计、档案、地方志、红十字等工作。

**实施健康安庆建设。**编制并落实“健康安庆2030”规划纲要。深化医药卫生体制改革，推进医疗、医保、医药“三医联动”，健全分级诊疗体系，实现县域医共体建设全覆盖。加强重大疾病防控，推进中医药事业发展，完善基层医疗卫生服务网络，打造30分钟基层医疗服务圈。加强妇幼保健能力建设，提高出生人口素质。推进城乡居家养老服务中心建设，完成城区127个居家养老服务站点改扩建。加强食品药品生产、流通、消费等环节监管，提高安全保障水平。加强群众健身活动场地和设施建设，构建市县乡村四级全民健身设施网络和城市社区15分钟健身圈；持续推进全民健康教育，广泛开展健康社区、健康村镇、健康单位、健康家庭创建活动。

**开展信用安庆建设。**积极创建国家社会信用体系建设示范城市，建成地方信用信息共享平台，建立信用红黑名单制度，持续开展金融环境整治，完成恶意逃废债、知识产权侵权等失信行为信用记录全收集，实现统一信用代码全转换，在政府采购、财政奖补等10个领域开展信用信息大数据示范应用，推行事前信用承诺、事中信用分类监管、事后信用联合奖惩制度，加快构建以质量为基础、以信用为核心的市场监管体系。

**谋划智慧安庆建设。**编制智慧安庆总体规划，优先启动全市统一的政务数据中心建设，逐步实现信息资源互联互通和共享。完成“宽带中国”示范城市创建任务，推进国家信息消费试点城市建设，开展电信普遍服务试点项目建设，提升农村及偏远地区网络基础设施水平，确保全市所有行政村的宽带接入能力均达到12兆以上。

三、努力提高政府工作水平

牢固树立“四个意识”特别是核心意识、看齐意识，坚决维护以习近平同志为核心的党中央权威，以新理念指导新实践，切实加强政府自身建设，全面提升工作效能，不断增强执行力和公信力。

**提高依法行政水平。**深入实施“七五”普法，完善政府学法制度，加强以《宪法》、行政法为重点的法治宣传教育培训，切实增强政府工作人员法治意识、法治思维和法治精神。配合市人大常委会做好《安庆市城市管理条例》、《安庆市电动车管理办法》等立法工作，加强城市建设管理、环境保护等领域的政府规章建设。深化行政执法体制改革，推进行政处罚权相对集中和综合执法，全面开展重大执法决定的法制审核和行政执法公示工作。自觉接受人大法律监督、政协民主监督，广泛听取民主党派、工商联、无党派人士和工会、共青团、妇联等人民团体的意见，切实做好人大议案和政协提案的办理工作。主动接受社会监督和舆论监督，让人民群众更有效地监督政府。

**增强创新发展能力。**创新发展理念，加强系统思维，切实做到谋定而后动，注意把时代要求、客观规律、群众期盼和现实条件有机地结合起来，以功成不必在我的胸怀，多做打基础、利长远的事。创新行政管理，深化“放管服”改革，完善权责和涉企收费清单制度，建立公共服务、中介服务清单，构建“亲”“清”政商关系，充分激发市场活力。创新公共服务，更多地运用市场化的办法，借助信息化的手段，调动起社会力量，解决公共服务供给不足、能力不够的问题。创新政策激励，认真落实“产业政策要准”的要求，及时修订出台产业扶持政策，打好政策“组合拳”，充分运用资金奖补、融资担保、股权投资等综合方式，将政府的公共资源从有限供给转向有效供给，实现政策效益最大化。

**切实转变工作作风。**坚持领导干部调查研究、定期接待群众来访、“1584”热线互动、群众满意度测评等制度，大力推行“一线工作法”，深入基层、深入企业，着力解决事关群众切身利益和企业发展的困难与问题。推进机关效能革命，全面加强政务服务和政务公开，拓展“互联网+政务服务”覆盖面，争创社会管理和公共服务综合标准化建设国家试点。完善目标考核办法，严格绩效管理和奖惩兑现，持续开展“整懒治庸”专项行动，强化责任担当，不断提高政府服务的效率和质量。

**扎实推进从严治政。**严格落实全面从严治党主体责任，全面加强和规范党内政治生活，持续提升政府系统从严管党治党水平。严格落实工作责任制和责任追究制，加大督查问责力度，做到有令必行、有禁必止，确保政令畅通。严格落实党风廉政建设责任制和“一岗双责”，加强行政监察，推进审计监督全覆盖，聚焦脱贫攻坚、征地拆迁、环境保护等重点领域，坚决查处和纠正侵害群众利益的不正之风。全面加强反腐倡廉制度建设，健全廉政风险防控机制，真正把权力关进“铁笼”、套上“紧箍”，着力营造风清气正的政务环境。

各位代表！干在实处永无止境，闯出新路重任在肩。让我们更加紧密地团结在以习近平同志为核心的党中央周围，在省委、省政府和市委的坚强领导下，振奋精神，扎实苦干，不断开创“四个强市”建设新局面，努力谱写人民美好生活新篇章，以优异成绩迎接党的十九大胜利召开!
